# 

**Published:** 1826-10-01

**Authors:** 


### BIBLIOGRAPHICAL RECORD;

OR,

*Works received for Review between the 15th of June and the 15th of September*, 1826.

1. The American Medical Recorder, for April, 1826. 8vo.

---

The following works have been presented by M. Bailliere, of Paris, and No. 3 Bedford-street, Bedford-square, London, viz.

2. Discours sur la Biologie, ou Science de la Vie, &c. 8vo. pp. 72. Paris, 1826.

3. Recherches sur les Cas d'Utérus Double et de Superfétation. Par A. L. Cassan, &c. &c. 8vo. pp. 84, avec une Planche, Paris, 1826.

4. Nouveaux Elemens de Pathologie Medico-Chirurgicale. Par Mess. Roche et Sauson. Tomes 2, 8vo. pp. 666, 711. Paris, 1826.

5. Mémoires de la Société Médicale d'Emulation de Paris. Tome neuvieme, avec deux Planches. 8vo. pp. 544. Paris, 1826.

6. Traité Clinique et Experimental des Fievres dites Essentielles. Par J. Bouillaud, M.D. &c. 8vo. pp. 552, Paris, 1826.

---

7. Transactions of the Medico-Chirurgical Society of Edinburgh. Instituted 1821. Vol. ii. with Plates. 8vo. pp. 411. Edinburgh, 1826.

8. An Introductory Lecture delivered in the College of Physicians and Surgeons, at the opening of the Winter Session, on the 1st November, 1825. By David Hosack, M.D. &c. 8vo. stitched, pp. 61. New York, 1825.

---

9. The Philadelphia Journal of the Medical and Physical Sciences. No. 5, New Series. Philadelphia, 1826.

---

10. Observations on the Medical Character. By David Hosack, M.D. &c. 8vo. stitched, pp. 38. New York, 1826.

---

11. An Introduction to the Practice of Midwifery. By Thomas Denman, M.D. Licentiate in Midwifery of the College of Physicians in London, &c. From the last London Edition, revised by the Author. With Notes and Emendations by John W. Francis, M.D. &c. 8vo. pp. 684, 16 plates. New York, 1825.

---

12. A Synopsis of the Diseases of the Eye, and their Treatment: to which are prefixed a Short Anatomical Description, and a Sketch of the Physiology of that Organ. By Benjamin Travers, F.R.S. Surgeon to St. Thomas's Hospital. With Notes and Additions by Edward Delafield, M.D. &c. First American, from the third London Edition. 8vo. pp. 474, with coloured plates. New York, 1825.

☞ *This reprint of Travers on the Eye is very creditable to our Trans-Atlantic brethren. The coloured plates which, we understand, are the first that have been executed in America, are really well finished, and would not shame our British Artists.*

---

13. A Treatise on Diet, with a view to establish, on Practical Grounds, a System of Rules, for the Prevention and Cure of Diseases incident to a Disordered State of the Digestive Functions. By J. A. Paris, M.D. F.R.S. Fellow of the Royal College of Physicians, &c. 8vo. pp. 307, London, 1826.

---

14. A System of Anatomical Plates, with descriptive Letter-Press. By John Lizars, F.R.S.E. Fellow of the Royal College of Surgeons, Corresponding Member of the Medical Society of Emulation of Paris, and Lecturer on Anatomy and Surgery, Edinburgh. Part X.—The Organs of Sense and Viscera. Folio, 8 plates, pp. of letter-press 66. Price 10s. 6d. plain; £1. 1s. coloured.

☞ *This number does, we think, full justice to Mr. Lizars' fame, and this is no mean praise. The different views of the human eye are clearly and beautifully executed; so are the delineations of the epidermis. The plates of the viscera, too, are correct and highly finished.*

---

15. A Description of the new patent instrument for extracting teeth; also of a patent method of fixing artificial teeth. By S. P. Delafons, Surgeon-Dentist. 8vo. pp. 76, with two plates. London, June 26th, 1826.

---

16. Anderson's Quarterly Journal of the Medical Sciences, for July, 1826.

☞ *In Exchange.*

---

17. Phrenology, in connexion with the study of Physiognomy. By G. Spurzheim, M.D. &c. Royal 8vo. pp. 192—and 34 Plates. Treuttle and Wurtz, London, June 1826. Price 22s. boards.

☞ *See the present Number, page 468.*

18. The Edinburgh Journal of Medical Science, No. 3, for July, 1826. Octavo pp. 248. Two plates. *(In exchange.)*

19. A short account of the Analization, Effects, and Application of the Mineral Springs of Kaiser Franzensbad, near Eger in Bohemia. By J. J. Hecht. 8vo. pp. 62. Callow and Wilson, London, 1826.

20. Remarks on a recent Effort to subvert the Charter of the Royal College of Surgeons; with animadversions on the Evil Tendency of "The Lancet;" and Observations respectfully addressed to General Practitioners, on the Means of Maintaining their Privileges and Respectability. By William Cooke, Member of the Royal College of Surgeons, Secretary to the Hunterian Society, Editor of an Abridgement of "Morgagni," &c. 8vo. sewed, pp. 89. Underwoods, 1826.

21. Observations on the Expediency of Instituting a Friendly Association of the Medical Profession throughout Scotland, for insuring a Provision during Sickness and Old Age, Widows' Annuities, Endowments to Children, &c. By Edward Duffin Allison, Surgeon. 8vo. sewed, pp. 36. Edinburgh, 1826.

22. Revue Medicale, Française et Etrangère, et Journal de Clinique, de l'Hôtel Dieu, et de la Charité de Paris. Juin, Juillet, et Aout, 1826. *(In Exchange.)*

23. Principles of Dental Surgery; exhibiting a New Method of Treating the Diseases of the Teeth and Gums; especially calculated to promote their health and beauty, accompanied by a general view of the present state of Dental Surgery, with occasional references to the more prevalent abuses of the art. In two parts, by Leonard Koecker, Surgeon-Dentist, Doctor in Medicine and Surgery; Member of the Medical and Linnæan Societies of Philadelphia, &c. 8vo. pp. 445. London, 1826.

☞ *This valuable work in our next.*

24. The North American Medical and Surgical Journal—Nos. I.—III. January and July. Philadelphia, 1826. *(In exchange.)*

25. The American Medical Review and Journal of original and selected Papers in Medicine and Surgery. Philadelphia, 1826. *(In exchange.)*

26. An Anatomical Drawing and Diagram of the Eye. By W. P. Cocks. London. Highley, 1826.

27. Dissertatio Medica Inauguralis de Phthisi Pulmonali. Auctore Dav. Jacobo Hall, M.D. Chirurgo Adjuv. in Classe Britannica. Edinburgh, 1826.

☞ *This little thesis evinces a very fair acquaintance with the best modern writers, and the latest researches on pulmonary consumption.*

28. A Treatise on a Modified Application of Moxa, in the Treatment of Stiff and Contracted Joints; and also in Chronic Rheumatism, Rheumatic Gout, Lumbago, Sciatica, Indolent Tumours, &c. &c. Illustrated by Cases and Plates; with Observations on the different Remedies hitherto employed in the Treatment of Diseased Joints; and an Investigation into the Nature, Causes, and Treatment of Spinal Diseases. Second Edition. By James Boyle, Esq. Surgeon to the Middlesex Infirmary, &c. 8vo. pp. 219. London, 1826 *(In our next.)*

29. Part Third of a Series of Elementary Lectures on the Veterinary Art: wherein the Anatomy, Physiology, and Pathology of the Horse are essayed on the general principles of Medical Science. By William Percivall, M.R.C.S. &c. 8vo. pp. 502. London, 1826.

30. A System of Anatomical Plates; with descriptive Letter-press. By John Lizars, F.R.S.E. &c. Part XI. Abdominal Viscera, together with the Male and Female Organs of Generation.

☞ *This fasciculus contains seven plates of exquisite workmanship. The first is occupied with the kidney, and the other six contain various and excellent views of the male and female organs of generation.*

*The accompanying descriptive part contains upwards of* 100 *pages of letter-press, and corresponds in ability of execution with the plates.*

---

For the following works we have to return thanks to a liberal foreigner (J. B. Balliere, Medical Bookseller, Bedford-street, Bedford-square,) who has settled here for the sale of foreign medical works, viz.

31. Calmeil (L. F.) de la Paralysie, consideré Chez les Alienées, et Recherches faite dans le Service de Feu, M. Roy, Collard, et Esquirol. En 8vo. prix 6s. 6d.

32. F. Tiedemann et L. Gmelin Recherches Experimentales sur la Digestion considerée dans les Quatres Classes d'Animaux Vertebrés. Traduites de Lallemand, par A. J. L. Jourdan. *Premiere* Partie. 1826. Prix 8s. La Seconde Partie paraîtra a la même Adresse dans deux mois.

33. Hurtrel d'Arboval, Dictionnaire de Medecine et de Chirurgie, Vétérinaires. Tom. 1, en 8vo, prix 8s. Les Tomes 2 et 3 paraîtront de 2 mois en deux mois.

34. (J. F.) Meckel, Manuel d'Anatomie, Générale, Descriptive, et Pathologique. Traduit de Lallemande, par Messrs. Jourdan, A. J. L. et G. Breschet, Chef des Travaux Anatomiques de la Faculté de Paris. 3 fort Volumes en 8vo. Paris, 1825. Prix £1. 5s.

35. Traité, Historique et Dogmatique, de la Taille. Par F. J. Deschamps, Membre de l'Institute, Chirurgeon-en-Chef de l'Hôpital de la Charité; avec un Supplement dans lequel l'Histoire de la Taille est continuée depuis la Fin du Siècle dernier jusq'a ce Jour. Par L. J. Begin, Doct. en Med. 4 Vol. en 8vo. prix £1. Paris, 1826.

36. Traité Clinique et Exprimentale des Fièvres dites Essentielles. Par J. Bouillaud, Doct. en Med. de la Faculté de Medecine de Paris. En 8vo. prix 7s Ann. 1826.

37. Traité Complet de l'Anatomie de l'Homme, comparée dans les Points les plus importants a celle des Animaux, et considerée sous le double Rapport de l'Histologie et de la Morphologie. Par Hyp. Cloquet, Professeur Agregé a la Faculté de Paris. En 4to. Paris, 1826.

---

38. A Comparative View of the more intimate Nature of Fever; deduced from Physiological Analysis, and illustrated by Critical Remarks and Practical Observations. By James Black, M.D. S.R.N. and Member of the Royal College of Physicians of London. 8vo. sewed, pp. 118, Price 4s. 6d. Longmans, September 12th, 1826.

☞ *In this little work there are some acute criticisms on various modern theories of fever, and an attempt to establish an eclectic doctrine, which is, at least, consonant with judicious practice.*

39. The Hunterian Oration, delivered before the Royal College of Surgeons, in London, on Tuesday, February the 14th, 1826. By SIR ANTHONY CARLISLE, Kt. F.R.S. Surgeon Extraordinary to His Majesty, &c. Dedicated by permission to Mr. Secretary Peel. Royal Quarto, pp. 47. London, Sept. 15th, 1826. Price 7s.

---

40. A Treatise on Repelling the Paroxysm of Intermittent Fevers; illustrated with Cases. By JOHN BROWN, M.D. &c. Boston, Lancashire, September 15th, 1826. 8vo. pp. 60. Underwoods, London.

---

41. A Conspectus of Prescriptions in Medicine, Surgery, and Midwifery, containing upwards of a Thousand Modern Formulæ, including the New French Medicines, and arranged Tables of Doses. Selected from the highest Professional Authorities, intended as a Remembrancer for General Practitioners. The Second Edition enlarged and improved. 12mo, pp. 216. Anderson, West Smithfield. 1826. Price 5s. sewed.

### *Additional Subscribers since last Quarter.*

Bonthron, Mr. Surgeon, South-Moulton street, Hanover-square
Campbell, Dr. Lecturer on Midwifery, Picardy Place, Edin.
Chirurgical Book Society, Chichester.
Clapton, Mr. Apothecary, King-Street, Westminster.
Clarke, John Fred. M.D. 51st Regiment.
Davis, Mr. J. B. Surgeon, Shelton, Staffordshire.
Dickson, R. Esq. Surg. H. M. Ship Raleigh, to Mediterranean.
Dill, Mr. John, Hon. E. I. C. Service.
Harrison, Dr. Edward Holles St.
Hitch, Mr. Surgeon, Tewksbury.
Hughes, Thomas, M.D. M.R.C. S. E. &c. Formerly House-Surgeon to the St. Mary-la-bonne Infirmary, &c. &c. Hereford-st. Park Lane, London.
Ingham, C. M.D. Surgeon, 29th Foot.
Irvine, Mr. John (B.) Surgeon, Royal Navy, Surgeon and Agent for Sick and Wounded, at Mooille.
Liddell, Mr. Assistant Surgeon, Bombay Establishment.
Medical Library, Corfu.
Ramsay, Mr. Surgeon, Pernambuco—S. America.
Rucco, Dr. Julius, late Professor of Anatomy to the Royal Medical College of Naples, Leicester Square, London.
Seaton, Mr. Surgeon, Bridge St. Westminster.
Sierra Leone Med. Book-Society.
Thorly, Thomas, Esq. Brighton.
Van Oven, B. Esq. Fenchurch-Buildings.
Weston, Mr. Student, Edinburgh.
Winterton, Mr. Surgeon, to South America.
Windsor, John, Esq. Manchester.
Winton, Dr. Hon. E. I. Company's Service.

---

*Changes of Residence.*

Dr. Marshall Hall, from Nottingham, to Keppel Street, Russel Square.
Dr. Hunt, from York Street, St. James's Square, to Richmond.

### INDEX.

A

Abraham, Mr. on the effects of civilization ... 265
Absorbents, inflammation of, from poisoned wounds ... 365
Absorption, experiments on its nature ... 321
Acetate of morphine externally, its good effects ... 252
Acupuncture, effects of, in a neuralgia ... 621
Alveolar processes of both jaws removed; a case ... 288
Ammoniacal pomade in cataract, its utility ... 179
Amputation of the lower-jaw, Dr. Mc. Clellan's case ... 532
Amputation of a toe, death from 578
Anatomy of leeches described .. 61
Andral, M. jun. on cicatrization of tubercles in the lungs ... 214
Aneurism of the aorta, Dr. Adam's case ... 43
Aneurism, Mr. Bell's case of ... 574
Aneurisms in the brain, M. Serres on the rupture of ... 249
Angina maligna, muriatic acid *topically* in ... 421
Angina maligna and croup, supposed identity of ... 422
Angina diptheritica, diagnosis of 425
Animal heat and respiration, Dr. Williams on ... 401
Annesley, Mr. his reports of Indian diseases ... 342
*Anonymous* school of medicine, alias the *idiopathists*, their errors, &c ... 226
Antimonial plaister, recipe for .. 291
Anus, leeches to the; remarks on the practice ... 53
Aphonia, periodical, a case of .. 525
Apoplexy, history and pathologyof 415
Army and navy practitioners, their general merits ... 210
Artificial anus successfullytreated, a case ... 292
Artificial anus after pregnancy .. 525
Associated Apothecaries, their report ... 630
Asthma, a case of ... 217
Asthma; antipathy of the French to the name ... 218

B

Barometers; leeches said to be such 59
Barras, Dr. his case of gastralgia 489
Barry, Dr. on the circulation of the blood ... 313
Beauprè, M. on the effects of cold 77
Bell, Mr. G. his cases of wounded nerves ... 245
Beriberi, Mr. Hamilton on ... 396
Black drop, *reminiscences* on the 291
Bladder, remarkable disease of .. 287
Bladder, spasm of the ... 502
Bladder, Mr. Thompson's case of distension of the ... 507
Bladder, living worms discharged from the ... 528
Bleeding in convulsions, Mr. North's sentiments on ... 158
Bleeding, Medicus on ... 232
Bourgeois, M. his case of scirrhus of the stomach ... 206
Brain, Dr. Clutterbuck's *primum mobile* in fever ... 7
Brain, its state in convulsions .. 153
Brain, Dr. Mill's observations on its diseases ... 350
Breschet, M. on a new kind of extra-uterine pregnancy ... 518
Bretonneau, Dr. and his theory of fever ... 199
Bretonneau, Dr. on "diptheritis" 419
Brodie, Mr. his theory of cold .. 93
Bronchitis, Mr. Jowett's case of .. 255
Bronchocele, some fatal cases of 189
Bronchocele, some cases of, treated with iodine ... 196
Bronchocele, thyroid arteries *tied* for ... 585
Broussaiism *v.* the errors of the schools, a case ... 21
Broussais, M. and our *leading* Doctors ... 30
Butel, the Chevalier de, on plague 233
Bye-law of College of Surgeons .. 633

C

Cæsarian section, another successful case ... 284
Calculus extracted from the female bladder by dilatation; two cases ... 402-3
Calcutta Medical Society, its Transactions ... 36

Calomel, its inapplicability to the French stomach ............ 74
Calomel, its *moderate* doses in the olden time ................ 295
Calomel *not* the cause of green stools ...................... 353
Cancer, antiphlogistic treatment of ....................... 286
Cancrum oris infantium, Dr. Coates on .................. 586
Cantharides, poisoning by, a case 240
Carter, Dr. his hospital reports.. 195
Cataract, Dr. Gondret's observations on.................... 177
Cataract in India, Mr. Richmond on .......................... 263
Cellular inflammation from poisoned wound................ 366
Cerebral and worm fever, parallel between.................... 219
Cerebral affection, Dr. Latham's case ........................ 577
Chloruret of lime, its antiseptic powers .................... 298
Cholera morbus, Mr. Montgomery's cases of .................. 259
Cinchonine, sulphate of, its employment .................... 283
Circulation of the blood, Dr. Barry's researches on the......... 313
Civilization, its influence on human life.................... 265
Clarke, Dr. on the fever of Smyrna 637
Clutterbuck's, Dr. theory of fever 1
Cold, M. Beaupré on its effects .. 77
Cold, theory of................ 79
Cold, its medicinal properties.... 89
Cold and the warm bath conjointly in convulsions .............. 159
Colica pictonum, its seat ...... 298
College of Surgeons, new regulations of ..................... 632
Collins, Dr. his case of eleven-months' pregnancy ........... 212
Colon, a case of stricture and perforation of.................. 275
Combustion, spontaneous, case of 286
Congenital blindness removed .. 622
Constantinople, curious singularity of the plague at.......... 237
Constitutional irritation, Mr. Travers on ..................... 95
Constitution, state of, amongst our students .................. 375
Contagion and infection, their ultimate identity............... 330
Contagion, specific, does it exist after death ?................ 378
Conversations on the "physiological doctrine".................. 1
Convulsions from loss of blood.. 119
Convulsions of infants, Mr. North on the ..................... 149
Coombe, Mr. his system of phrenology ...................... 437
Croup ; exudation expelled from the larynx.................. 538
Cupping in poisoned wounds strongly recommended......... 38
Cupping-glass in poisoned wounds, its application .............. 331
Cutaneous disorders, treatment of, at the Hop. St. Louis ........ 76
Cutaneous medication, M. Lesieur on...................... 531

D

Death, sudden, from peritoneal inflammation ................ 276
Delirium tremens, Mr. Playfair's paper on .................. 40
Dieffenbach on Staphyloraphy .. 188
Diet, certain *dicta* respecting, remarks on ..................... 103
Digestive organs, nature of the sensibility of the............... 27
Dill, Dr. his dissertation on cuticular absorption ............. 240
"Diptheritis," Dr. Bretonneau on 419
Disease, *morbid* generalizations of 351
Disease of the heart, Dr. Macmichael on................... 605
Disease of the heart, remarkable cases of ..................... 623
Dissection-wound, death from .. 364
Dissection-wound, severe case of 371
Dissection-wound, fatal case of.. 615
Distended bladder ; Mr. Thompson's case .................. 507
Dothinenteritis—another " new theory of fever!" ........... 199
Dracunculus, mercurialization for 43

E

Edinburgh Medico-Chir. Transactions ..................... 392
Egyptian mummies, Dr. Granville on ........................ 46
Eleven-months' pregnancy, Dr. Collins' case .............. 212
Emphysema post partum, a case of 287
Empyema, a case of, cured by paracentesis ................. 267
Enteritis, instructive case of .... 202
Epilepsy in infants, history and treatment of................ 163

Epilepsy, tartar-emetic ointment in ........................ 195
Epilepsy and mental alienation, their nature ................ 229
Epilepsy from tænia, two cases of 538
Epilepsy, some cases of ........ 358
Erysipelas, contagious, Dr. Stephenson's cases of ............ 404
Examen des doctrines medicales, notice of it.................. 1
Exanthematous ophthalmia, Mr. Wardrop on ................ 393
Extirpation, successful, of a scirrhous parotid ............... 223
Extract of garden lettuce, Dr. François on ................ 522
Extra-uterine fœtus successfully removed .................. 275
Extra-uterine pregnancy, new kind of ...................... 518
Extra-uterine conception, case of 529
Extra-uterine conception, Dr. Doudement on................ 618

F.

Fat and blood, vomited, a remarkable case.................. 247
Fawdington, Mr. his case of melanosis .................... 335
Femur; supposed case of partial fracture of it................ 249
Fever, an inquiry into its seat and nature .................... 1
Fever, idiopathic, its onset and progress ...................... 9
Fistulæ, M. Roux's mode of treating........................ 594
Follicular ulceration, Dr. Hewett on ........................ 557
Fracture of the spine, M. Lisfranc on ....................... 560
Fractures and contusions, severe cases of.................... 115
French *pratique*, Dr. Scudamore's ideas on the .............. 74
French criticism, or vive la *grande nation!* ................ 253

G

Gangrene from cold.............. 84
Gardner Peerage case, report on 170
Gastralgia mistaken for gastritis, Dr. Barras on ............... 489
Gastralgia, some cases of ...... 493
Gastralgia and gastritis, diagnosis of ........................ 496
Gastro-enteritis, progress of, &c. 25
Glanders, the human subject infected by the matter of, some cases ...................... 280
Gondret, Dr. on cauterization in cataract .................... 177
Graduates of Scotland—degraded in England ................ 636
Granville, Dr. on mummies .... 46
Granville, Dr. on gastrotomy.... 576
Gregory's, Dr. Practice of Physic 408
Guilbert, M. on leeching the os uteri ...................... 508
Gullet, Dr. Monro on the spasm of ........................ 500
Guthrie, Mr. on phlebitis ...... 562

H

Hæmoptysis, employment of cold in ........................ 93
Hæmorrhage from leech-bites .. 57
Hæmorrhage and suppuration, cases of .................... 125
Hepatitis, its insidious nature .. 347
Hernia, cases of, by Messrs. Shaw and Boyle ................ 569
Herpes Zoster, lunar caustic in.. 573
Hewett, Dr. on follicular ulceration .................... 557
Hooping cough, Dr. Gregory's opinions on ................ 409
Hooping cough, Dr. Dawson's opinions on ................ 413
Hospital reports, Dr. Carter's.... 195
Hospital reports, Dr. Bland's.... 567
Hospital reports, by M. Lisfranc 570
Hour-glass contraction of the bladder ...................... 503
Howship, Mr. his case of mollities ossium .................. 405
Human pregnancy, evidence on the duration of.............. 170
Hunger-cure successfully employed .................... 527
Hutchison, Mr. on imperforate anus .................... 511
Hydrocephalic effusion, is it removeable? .................. 357
Hydrocephalus, some cases of .. 355
Hydrochloric acid, its powers in diptheritis................ 429
Hydrophobia simulata, curious case of .................. 261
Hydrophobia, doubtful case of .. 564

I

Ictus solaris, a case of, by Mr. Brown .................. 290

Imperforate anus, successful operation for .............. 187

Imperforate anus, Mr. Hutchison on ........................ 511
Imperforate anus,various cases of 515
Imperforate rectum,caseof, cured 615
Infanticide, alleged case of...... 239
Infanticide, new tests for the detection of .................. 297
Infanticide,question of insanity in 617
Infants, their natural language.. 150
Inflammation from injuries and operations ................. 121
Injuries of the head, Mr. Jeffreys on ........................ 599
Injuries of the thorax,Messrs. Bell and Shaw on................. 625
Insanity, idiopathic and symptomatic ...................... 226
Intemperance, melancholy consequences of ; a case .......... 361
Intermittents, an *eye-sore* to the *exclusionists* ................ 11
Intermittents, Broussais *v.* bark 29
Intestinal ulcers, cicatrization of 192
Intestinal tube,spasm of the, case 504
Inverted toe nail,M. Dupuytren on 575
Iritis, chronic, Mr. Watson on .. 400
Iron-filings in cases of poisoning 611
Irritability, proper and sympathetic ......................... 97
Irritation, direct, Mr. Travers' remarks on .................. 110
Irritation of the body, its effects on the mind ................ 388
Issue on the vertex capitis in epilepsy ...................... 360

J & K

John Hunter and M. Anel compared ! ...................... 254
Johnson, Dr. Rawlinson, on the leech ...................... 52
Jowett, Mr. his case of empyema cured by paracentesis......... 267
" Kirronosis," M. Lobstein on .. 517

L

Lanza, professor, his new medical logic ...................... 128
Larrey, Baron, his treatment of traumatic erysipelas ........ 205
Leech, Drs. Johnson and Price on the...................... 52
Leech, diseases of the, and their prevention .................. 66
Leech-importers, their enormous receipts.................... 72
Leeches, Dr. Pallas on the management of ................ 289
Licentiates of the College of Physicians, their degradation .... 636
Linea alba, rupture of ; a case .. 242
Lisfranc, M. on White Swelling.. 546
Lisfranc on fracture of the spine 560
Lisfranc, his hospital reports.... 570
Lithontripteur, success of, in France ..................... 536
Lithotomy inducing mania, a curious case ................. 114
Lithotomy, some hints on ...... 118
Local injury, its effects on the constitution ................ 104
Louis, M. his case of subacute metritis, &c.................. 244
Lunar caustic, Mr. Higginbottom's paper on................ 260
Lungs,wound of the,a severe case 204
Lungs, a case of sudden congestion of the.................. 292
Lusus naturæ, two instances of.. 399

M

Mackenzie, Mr. and Dr. Bretonneau, their claims............ 436
Mania, curious and fatal case of.. 203
Marshes communicating with the sea, M. Georgini on.......... 262
Masked cerebral affection, case of 584
Medical etymology, curious samples of...................... 303
Medicine, *uncertain* no longer.. 21
Medico-Chirurgical Transactions of Edinburgh ............... 392
Medico-chirurgus, his complaint, 209
Medullary and cortical parts of the brain in epilepsy and insanity...................... 231
Melanosis of the stomach ; M. Andral, jun................. 244
Melanosis, Mr. Fawdington's case of ......................... 335
Mental alienation, prize essays on 226
Mercury, its powers in chronic and acute diseases ........... 348
Mercury in diptheritis........... 430
Metritis, with inflamed veins, a case ....................... 242
Mills, Dr.on disorders of the brain 350
Mind, its influence on the body.. 17
Mitchell, Mr. his case of wounded lungs ...................... 204
Mollities ossium, a melancholy case of...................... 405
Moral agents, excluded from medicine! .................... 227
*Mummification*, Dr. Granville's recipe for ................... 50

Mummy, what presented itself on opening one .................. 49
Museum, Hunterian, observations on ........................ 635

N

Nature, disease, and the doctor at issue ........................ 34
Neuralgia of the scalp cured by operation .................... 295
Neuralgia, Dr. Trevor on ...... 535
Neuralgia, a curious case of .... 612
New medical logic, Vincenzo Lanza's .................... 128
New medical logic, Vincenzo Lanza's .................... 470
Nitrous acid and opium, in dysentery ........................ 580
North, Mr. on the convulsions of infants .................... 149
Nose, loss of, in Russia ........ 523

O

Obstinate vomiting, cured by extract of marigold............ 197
Œsophagus, curious case of stricture of the.................. 294
Oil in the blood, Dr. Adam's case of ........................ 39
Opiates in convulsions, Mr. North's remarks on .................. 160
Os uteri, M. Guilbert on the leeching of the.............. 508
Ovaria, operations on, in Kentucky .................... 620

P

Palpitatio cordis, digitalis in.... 277
Papillæ of the tongue, Dr. Ravin on ........................ 288
Paracentesis successful in empyema .................... 267
Paralysis, some rare cases of .... 602
Parcentesis thoracis, American case of .................... 582
Paris, Dr. on diet.............. 636
Parotid, scirrhous, extirpation of a 223
Partial fracture of the femur.... 249
Parturition, difficult case of.... 198
Pathology of fever ............ 11
Pericarditis, M. Louis on ....... 504
Pericarditis, Dr. Louis' diagnosis of ........................ 523
Periscope, preface to the........ 486
Pett, Dr. his melancholy case.... 368
Phagedenic ulcer, Mr. Babington on ........................ 591
Phlebitis, Mr. Guthrie on ...... 563
Phlegmonous erysipelas, tight bandage in.................. 554
Phrenology, Mr. Coombe's system of ........................ 437
Phrenology and physiognomy, Dr. Spurzheim on .............. 468
Physic, elements of the practice of, by Dr. Gregory .......... 408
Physician, answer to a letter from 635
Physicians, dissensions among .. 636
Placental presentation, a fatal case 614
Plague, isolation in, its effect.... 235
Plague, the Chevalier de Butel, on ........................ 253
Plethora and cause of convulsions 156
Ploucquet, the Clutterbuck of yore ........................ 15
Poisoned wounds, the cupping-glass in .................... 322
Poisoned wounds, Mr. Travers on 363
Polyphagia, M. Beaudè's case of.. 596
Popliteal aneurism, Mr. Allan ... 257
Predisposition to convulsions.... 151
Pregnancy, application of the stethoscope in .............. 607
Prostration of the system after injuries .................. 389
Pulmonary exhalation, experiments on .................. 612
Purgation, its frequent bad consequences .................. 31

R

Regulations of the College of Surgeons .................... 632
Reports of Indian diseases, Mr. Annesley's ................ 342
Respiration in the new-born child, Mr. Jowett on .............. 610
Retention of urine, *ingenious* mode of treating by *boring* the pubes!!.................. 285
Rheumatism of the Joints, Dr. Chambers on .............. 566
Rheumatism of the heart cured by acupuncture .............. 619
Richerand, M. and his *anti-gallican* liberality .............. 253
Richmond, Mr. on sea-water in ophthalmia .............. 223
Richmond, Mr. on cataract in India .................... 263
Roux, M. his surgical reports.... 593
Russian campaign; melancholy picture of it .............. 81

S

Scirrhus of the stomach, symptoms of .................... 208
Scirrhus, M. Lisfranc's report on 598
Sea-sickness, some hints on .... 251
Sea-water in ophthalmia, Mr. Richmond on ................ 222
Sloughing ulceration, Mr. Travers' cases of................ 552
Sloughing Ulceration, Messrs. Green and Rose on .......... 553
Smyrna, remarkable fever there 637
Spasm of muscular tubes, Dr. Monro on ................. 500
Spasmodic "crooping noise" in children; remarks on it....... 165
Specific diseases and medicines, Mr. Allan on................ 541
Spleen, rupture of in fevers.... 283
Spontaneous combustion, M. Hellis' case of.................. 543
Spurgin, Dr. his case of poisoned wound ..................... 370
Spurzheim, Dr. on phrenology and physiognomy............ 468
Squinting, modes of obviating .. 161
Staphyloraphy, Dr. Dieffenback's improvements ............... 188
Stethoscope, Dr. Scudamore on its use, &c................ 73
Stomach, perforation of by a bullet, a case................ 38
Stomach, case of scirrhus of .... 206
Stomach of a Broussaian, its marvellous properties............. 500
*Supplantation*, art of; the most approved method.......... 211
Suction, employment of in paracentesis thoracis ............ 273

T

Tartar emetic, bark as an antidote for................... 285
Testicle, hydatid, Mr. Brodie's case of .................... 595
Thoracic disease, severe case of.. 290
Throat, leeches in the.......... 54
Thyroid arteries tied for bronchocele ..................... 585
Tic-douloureux, cured by caustic, 528
Trachea, great compression of by a bronchocele ............... 191
Tracheotomy, some successful cases of ................... 433
Transactions of the Calcutta Medical Society................. 36
Transfusion, two cases of ...... 613
Traumatic erysipelas, actual cautery in ..................... 205
Travers, Mr. on constitutional irritation .................... 95
Travers, Mr. on poisoned wounds 363
Troillet, M. on the healing of intestinal ulcers .............. 192
Tubercles in the lungs, cicatrization of .................... 214
Tuberculated peritoneum, a case of........................ 285

U

Urethral stricture, observations on ......................... 570
Urinary calculus extracted from the female.................. 597
Uterine hæmorrhage, novel treatment of.................... 632
Uterus; inflammation of it, causing mania................. 203
Uterus, rupture of in early pregnancy; a case .............. 274
——— leeches to the mouth of the ........................ 508

V

Vagitus uterinus .............. 524
Variolous pustules, cauterization of ........................ 285
Vincenzo Lanza's New Medical Logic ..................... 470
Viper, experiments on the bite of the....................... 327
Vivisections and experiments, defence of .................. 314
Vomiting, obstinate, cured by acetate of morphine............ 252

W

Wardrop, Mr. on the exanthematous ophthalmia ............ 393
Wardrop, Mr. case of congenital blindness ................. 622
White-swelling, M. Lisfranc on.. 546
Worm fever, parallel between it and the cerebral ............ 219
Wounded nerves, Mr. G. Bell's cases ...................... 245

